# Inhibition of collagen fibril formation

**DOI:** 10.1186/1755-1536-5-S1-S29

**Published:** 2012-06-06

**Authors:** Andrzej Steplewski, Andrzej Fertala

**Affiliations:** 1 Department of Orthopaedic Surgery, Jefferson Medical College, Thomas Jefferson University, Philadelphia, PA, 19107, USA

## Abstract

**Background:**

The overall aim of presented study is to test the inhibition of the formation of collagen fibrils as the novel approach to reduce accumulation of pathological fibrotic deposits. The main hypothesis is that by interfering with the initial steps of the extracellular process of collagen fibril formation, it is possible to reduce the formation of fibrotic tissue.

**Methods:**

The experimental model includes antibody-based inhibitors that specifically bind to the sites that participate in the collagen/collagen interaction.

**Results:**

Employed antibody-based inhibitors effectively limit the amount of collagen fibrils formed *in vitro *and in engineered tissue models of localized fibrosis.

**Conclusions:**

(i) Inhibition of collagen formation is an attractive target to reduce excessive formation of fibrotic tissue.

(ii) Antibody-based inhibitors of collagen fibril formation are promising therapeutic agents with a potential to limit localized fibrosis in a number of tissues.

## Background

### Collagen self-assembly

**Figure 1:**
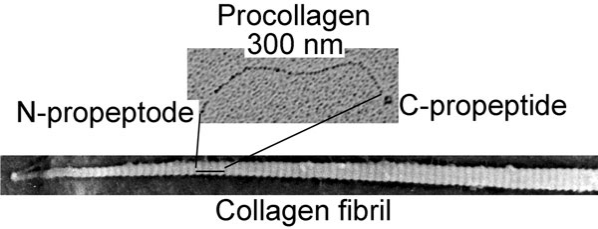
**Electron microscopy of a single procollagen molecule and a collagen fibril formed *in vitro *by self-assembly of collagen molecules**. A procollagen molecule, 300 nm in length, consists of the triple-helical domain flanked with the N propeptide and the C propeptide. Upon cleavage of the propeptides by specific enzymes, collagen molecules self-assemble to form collagen fibrils.

**Figure 2:**
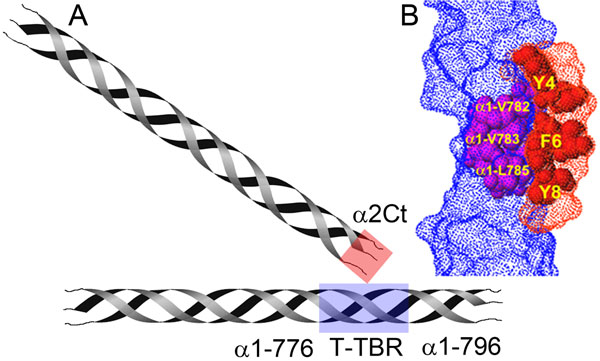
**Collagen/collagen interaction sites involved in fibril formation**. A, A schematic of collagen molecules interacting during fibril formation. Sites that are involved in collagen/collagen binding are indicated with red and blue boxes. These sites include the T-TBR (α1 776-796 fragment) and the α2Ct. B, A computer model representing interacting domains of two collagen molecules. The triple-helical fragment of the T-TBR is indicated in blue, while the fragment of the α2Ct of the interacting partner collagen molecule is indicated in red. Amino acid residues most likely engaged in collagen/collagen interaction are also indicated.

**Figure 3:**
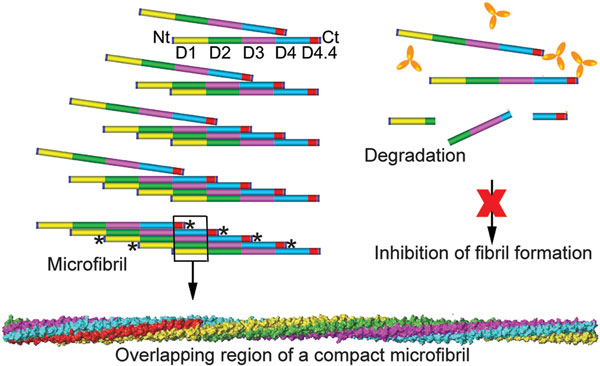
**Collagen/collagen interaction sites involved in fibril formation; the basic concept of inhibiting collagen fibril formation**. A, the stages of normal fibril formation by site-specific interaction between telopeptides of one collagen molecule and the T-TBR of the interacting molecule located in the D4 period; fibril-incorporated collagen molecules are very resistant to enzymatic degradation. B, by blocking the sites of critical collagen-collagen interaction, formation of fibrils is inhibited, preventing the accumulation of fibrils and allowing rapid degradation of excess collagen molecules. Asterisks represent sites of cross-links formation. The model of a microfibril illustrates the compact packing of collagen molecules (indicated in colors matching those for specific D periods) and indicates how the binding of a bulky inhibitor to collagen molecules would prevent such a compact organization.

Collagen I is the most abundant structural protein of connective tissues such as skin, bone, and tendon. This protein is first synthesized as a precursor molecule, procollagen, that is characterized by the presence of a rod-like central triple-helical domain flanked by short linear telopeptides and globular N-terminal and C-terminal propeptides [1]. Single procollagen molecules are the building blocks for the biologically-and mechanically-relevant collagen fibrils (Figure 1). The formation of collagen fibrils is initiated by enzymatic cleavage of N-terminal and C-terminal propeptides. The N-terminal propeptides are cleaved by a group of enzymes that includes a disintegrin and metalloprotease with thrombospondin motifs (ADAMTS)-2, -3, and -14, while the C-terminal propeptides are processed by the metalloprotease bone morphogenetic protein 1 (BMP-1) and by the other members of a closely related family of mammalian tolloid-like metalloproteases [2-4]. Such removal of procollagen propeptides exposes telopeptides, which drive collagen self-assembly by engaging in site-specific intermolecular interactions [5] (Figure 1, Figure 2, and Figure 3A).

### Collagen and fibrosis

In physiological conditions homeostasis of tissue collagens is constantly maintained, but during a number of pathological processes, the balance is shifted toward fibrosis, a process of excessive collagen production and accumulation. Fibrosis is a reactive process modulated by various factors propagated by an inflammatory tissue reaction. These factors trigger the local expansion of resident fibroblast subpopulations, modulate anabolic and catabolic processes taking place in these cells, and influence reactions governing the biosynthesis and degradation of the connective tissue components. Contributing to the metabolic modulation of the biosynthesis and degradation of collagenous proteins are cytokines and growth factors, a group of diverse molecules derived from blood cells, such as platelets, or elaborated locally by mesenchymal and epithelial cells [6].

Localized fibrotic reactions are quite common and frequently develop as a consequence of trauma or surgical procedures. For instance, after surgery of the abdomen, the formation of excessive scar tissue around abdominal organs, such as the intestines, can interfere with the function of such organs and may cause severe pain and even death. Another situation where excessive scar formation presents a major complication is in the eye after glaucoma surgery performed to create a pressure-maintenance valve. Frequently, however, excessive scar formation closes this pressure-reducing valve, thereby forcing the intraocular pressure to rise [7]. Moreover, excessive scarring of the vocal folds may severely alter their ability to vibrate, thereby causing a number of voice disorders [8].

### Inhibitors of fibrosis

At present, several biological processes critical for the development of fibrotic lesions are considered potential targets for inhibitors of fibrosis. These inhibitors aim at (i) reducing inflammatory processes associated with fibrosis, (ii) inhibiting biological functions of cytokines and growth factors that promote fibrosis, (iii) reducing cell proliferation, and (iv) decreasing the biosynthesis and enzymatic processing of procollagen molecules. The common characteristic of the above approaches is that they target broad upstream processes of the fibrotic cascade. Since most of these processes are involved not only in pathological fibrosis, but also in a number of physiological events, interfering with them is frequently associated with adverse effects [9-13]. Below is a brief characteristic of various clinical and experimental approaches employed currently and in the past to reduce localized fibrotic changes.

### Inhibition of the inflammatory phase

Inhibition of excessive fibrosis at the inflammatory stage has been widely investigated, and at present, is the most established approach to scar management. The inflammatory response can be regulated before, during, and after synthesis of the inflammatory mediators. The most commonly applied anti-inflammatory agents targeting production of inflammatory proteins are glucocortico-steroids. These compounds are effective in some cases of liver and pulmonary fibrosis and also in some patients treated for severe hypertrophic scars of the skin [11,14-17]. Because of the adverse side effects of steroids, particularly when applied systemically, their use is limited in chronic fibrotic diseases. Most nonsteroidal anti-inflammatory drugs studied as antifibrotic agents were reported to be ineffective [18].

### Inhibition of cytokines

A number of cytokines and growth factors have been identified in fibrotic processes, and their roles have been associated with the activation of the expression of genes encoding collagens and other structural macromolecules. Among these factors, members of the TGF-β family, connective tissue growth factor (CTGF), platelet derived growth factor (PDGF), and epidermal growth factor (EGF) play key roles. Perhaps the most attractive target for inhibiting fibrosis at the level of cytokines is TGF-β1. As demonstrated by Shah *et al*., injecting anti-TGF-β1 antibodies into margins of healing wounds significantly decreases scar formation [13]. TGF-β, however, has normal functions that make chronic administration of any inhibitor that indiscriminately blocks TGF-β activity problematic due to unwanted side effects. Moreover, the overall enthusiasm about using an anti-TGF-β1 approach to treat fibrosis is hampered by evidence that TGF-β1 may also function as a tumor suppressor [19,20]. The main concern, then, is that long-term exposure to TGF-β1 inhibitors could cause dangerous side-effects in the form of cancers. Still, more recently approaches targeting TGF-β1 via blocking connexin-mediated mechanisms were proposed. Specifically, the studies showed that cell-permeable peptides that block intracellular interactions of connexin 43 alter TGF-β1 functions, thereby limiting production of collagen in an animal model [21]. A clinical value of this intracellular approach, however, has not yet been examined.

In addition to targeting TGF-β1 and other cytokines at the protein level, attempts were made to inhibit the expression of the corresponding genes by antisense oligonucleotide or RNA interference (RNAi) approaches [22]. Although antisense oligonucleotide technology has been around for about two decades, it appears that, for a variety of reasons, this technology did not find a broad clinical use and it is primarily employed as a research tool.

### Inhibition of cell proliferation

Mitomycin-C and 5-fluorouracil inhibit the proliferation of cells by blocking DNA synthesis and transcription. A single application of these agents by injection was shown to be effective in reducing the recurrence of keloid formation after surgery. Injection of 5-fluorouracil, however, causes considerable pain, thereby limiting the use of this agent for keloid reduction [12,23-25].

### Inhibition of collagen synthesis

Because prolyl-4-hydroxylase-mediated post-translational modifications are required for the correct folding of individual pro-αchains into functional triple-helical molecules and for the secretion of such folded molecules into the extracellular space, this enzyme was identified as a potential target to limit collagen biosynthesis. For instance, "Doxorubicin", a commonly used chemotherapeutic agent that irreversibly inactivates prolyl-4-hydroxylase in human skin fibroblasts, inhibits collagen chain assembly. It is not clear, however, if this agent would be beneficial to patients with excessive scar formation.

Yet another approach to limit the production of "functional" collagen molecules was to administer proline analogues [26]. Because proline is a major residue in fibrillar collagens and constitutes ~10% of the total pool of collagen amino acid residues, the rationale behind this concept was that incorporating proline analogues to newly synthesized collagen chains, instead of native proline residues, would lead to the formation of non-functional collagen molecules. Although a number of proline analogues were developed a few decades ago, to date, they have not been used due to significant toxic side effects [26].

### Inhibition of procollagen-processing enzymes

One of the critical steps that involved in collagen fibril formation is the enzymatic cleavage of procollagen propeptides with procollagen N-proteinase (PNP) and BMP-1 [27,28]. The discovery of the amino acid sequence of BMP-1 prompted the research on its potential inhibitors [4,29,30]. The rationale of this approach was that inhibiting the cleavage of the C-terminal propeptides by BMP-1 would prevent fibril formation, thereby limiting excessive fibrosis. Several inhibitors of BMP-1 (*e.g*. acidic dipeptide hydroxamate) have been reported to be active *in vitro*, but their effect on preventing collagen fibril formation is not known [9]. An additional significant problem is that BMP-1, in addition to procollagens, processes a number of other macromolecules central to various important biological events not related to collagen fibril formation [31-35]. Moreover, BMP-1 knockout experiments demonstrated the presence of normal collagen fibrils in tissues of BMP-1-knockout mice, indicating that BMP-1 is not the only enzyme able to process procollagen *in vivo*, making it an unattractive target for inhibitors of fibrosis [35]. Similarly, PNP knockout experiments demonstrated the presence of fully processed collagen I and collagen II in tissues of experimental mice. Moreover, PNP knockout caused sterility in males, an unexpected adverse effect [36].

### Inhibition of formation of fibril-stabilizing cross-links

In physiological conditions, the chemical cross-linking of collagen molecules incorporated in collagen fibrils is critical for the mechanical stability of fibrils. Moreover, the presence of chemical cross-links makes fibril-incorporated collagen molecules more resistant to proteolysis. This notion is supported by a study which demonstrated that long-term stability of free collagen molecules at 37°C is quite low [37]. Because of such instability, exposed individual α chains of these molecules are readily accessible to proteases [37].

Formation of cross-links is an enzymatic process catalyzed by lysyl-oxidase. Since lysyl oxidase is a copper-dependent enzyme, it has been proposed that the use of copper chelators, such as D-penicillamine, could result in reduced cross-link formation, thereby limiting tissue fibrosis [38]. Postulations have also been made to employ β-aminopropionitrile, a compound that inhibits enzymatic activity of lysyl oxidase by irreversible binding to this enzyme [38,39]. In a clinical context, however, inhibitors of lysyl oxidase are not suitable for the treatment of fibrotic diseases because of their considerable toxicity.

## Discussion

### A novel concept; limiting tissue fibrosis by direct targeting collagen fibril formation --

Because of a number of limitations of current approaches to reduce excessive formation of localized fibrotic deposits, Chung *et al*. have proposed a novel concept. The premise of this new concept is that, regardless of etiology, fibrotic deposits are built primarily from collagen, specifically collagen fibrils. Consequently, Chung *et al*. have proposed a plan to inhibit formation of fibrotic deposits by limiting formation of collagen fibrils (Figure 2 and Figure 3) [40].

In contrast to the approaches presented above, the method proposed by Chung *et al*. targets a specific downstream event in a fibrotic cascade. Specifically, in this novel approach, the main target is the collagen/collagen interaction, a key process that drives collagen fibril formation (Figure 2, and Figure 3). The proposed target for inhibiting formation of collagen fibrils is well defined. It has been determined that critical collagen/collagen binding is mediated through interaction of the C-terminal α1(I) and α2(I) telopeptides (α1Ct and α2Ct) of one collagen molecule and the **T**riple-helical **T**elopeptide-**B**inding **R**egion (T-TBR) of another binding partner (Figure 2). It has been also determined that the T-TBR corresponds to the α1 776-796 fragment of collagen I (Figure 2) [41]. Note: numbering of amino acid residues considers the first glycine residue in a collagen triple-helical region as number "1".

Even though collagen fibrils *in vivo *are complex heterotypic structures consisting not only of collagen I, but also of other collagen types, such as collagen III and collagen V, it is predicted that the novel approach targeting collagen I self-assembly will still be effective. This notion is justified because collagen I contributes the most to the total protein mass of fibrotic tissue and other collagen types are most effectively incorporated into a fibril only when collagen I core is present [42]. Thus, by blocking the self-assembly of collagen I, it will be possible to interrupt the entire cascade of heterotypic fibril formation.

The prospect of the novel concept of inhibiting collagen fibril formation by blocking collagen/collagen binding for its clinical utility is supported, in part, by earlier reports on the inhibition of intermolecular interactions. For instance, the formation of amyloid fibrous deposits in Alzheimer's disease can be partially inhibited by a fragment of collagenous Alzheimer amyloid plaque component (CLAC), which blocks binding between full-length CLAC molecules [43]. Moreover, a number of therapeutic agents that act through blocking intermolecular interactions already exist in the clinical practice. Those agents include "Etanercept" (a fusion protein that neutralizes soluble TNF-α in psoriasis, rheumatoid arthritis, and psoriatic arthritis) and "Enfuvirtide" (a synthetic peptide that acts extracellularly and inhibits HIV entry into T cells). These fundamental similarities among approaches to inhibit various intermolecular interactions provide a valid point in support of targeting collagen/collagen interaction to limit excessive formation of collagen deposits.

### Strategies of designing inhibitors of collagen fibril formation

The original concept and the initial results on inhibiting collagen fibril formation as a way to reduce the formation of fibrotic deposits in a keloid-like model were described by Chung *et al*. [40]. In this study, the authors have demonstrated that a monoclonal antibody that binds to the α2 C-terminal telopeptide of human collagen I (anti-α2Ct) significantly reduces the amount of collagen deposited in keloid-like constructs formed subcutaneously in nude mice. The authors have concluded that this reduction was a result of blocking the α2 C-terminal telopeptide-mediated collagen/collagen interaction (Figure 2) [40]. Based on the above results, utilizing antibody-based inhibitors is, at present, a leading concept in an approach to reducing localized fibrosis through interfering with collagen fibril formation. Antibody-based approaches include engineering clinically-relevant IgG and scFv variants.

## Conclusions

-Inhibition of collagen formation is an attractive target to reduce excessive formation of fibrotic tissue.

-Unlike canonical inhibitors of excessive accumulation of collagen-rich deposits, inhibition of collagen fibril formation targets a specific, extracellular event.

-Blocking collagen fibril formation may serve as an independent or supporting method to reduce excessive, localized accumulation of collagen-rich deposits.

-Antibody-based inhibitors of collagen fibril formation are promising therapeutic agents with a potential to limit localized fibrosis in a number of tissues.

## Competing interests

The authors declare that they have no competing interests.

## Authors' contributions

AS carried out collagen inhibition studies. AF conceived of the study, participated in its design and coordination, and drafted the manuscript. All authors read and approved the final manuscript.
